# Safety and Immunogenicity of a *Klebsiella pneumoniae* Tetravalent Bioconjugate Vaccine (Kleb4V) Administered to Healthy Adults: A First-in-Human Phase I/II Randomized and Controlled Study

**DOI:** 10.1093/infdis/jiaf600

**Published:** 2025-11-25

**Authors:** C Alaimo, N Karaky, R Lawrence, E Bownes, S Haffner, M Kowarik, D Goldblatt, P Martin

**Affiliations:** LimmaTech Biologics AG, Schlieren, Switzerland; Infection, Immunity and Inflammation, Great Ormond Street Institute of Child Health, University College London, London, United Kingdom; Infection, Immunity and Inflammation, Great Ormond Street Institute of Child Health, University College London, London, United Kingdom; Infection, Immunity and Inflammation, Great Ormond Street Institute of Child Health, University College London, London, United Kingdom; Nuvisan, Neu-Ulm, Germany; LimmaTech Biologics AG, Schlieren, Switzerland; Infection, Immunity and Inflammation, Great Ormond Street Institute of Child Health, University College London, London, United Kingdom; LimmaTech Biologics AG, Schlieren, Switzerland

**Keywords:** *Klebsiella pneumoniae*, O antigen, vaccine, first-in-human trial, safety

## Abstract

**Background:**

Safe and effective vaccines are urgently needed to prevent *Klebsiella pneumoniae* infections. We assessed safety and immunogenicity of a tetravalent bioconjugate vaccine Kleb4V, containing O antigen-polysaccharides of *K. pneumoniae* serotypes (O1v1, O2a, O2afg and O3b).

**Methods:**

In this observer-blind, randomized, placebo-controlled, phase I/II trial (ClinicalTrials.gov NCT04959344), 166 healthy adults (16 aged 18–40 and 150 aged 55–70 years) were enrolled and randomized to receive 2 intramuscular injections of Kleb4V (16 or 64 μg of total O antigen with or without adjuvant AS03) or placebo on days 1 and 57. While the primary outcome was safety, the secondary outcomes included vaccine antigen immunogenicity.

**Results:**

Kleb4V was well tolerated, with most solicited and unsolicited AEs of mild to moderate intensity. Kleb4V was immunogenic for all four vaccine-serotypes at both doses. O1v1, O2a and O2afg specific IgG increased after the 1st vaccination and IgG persisted at six months after the second vaccination. For three of the four vaccine-serotypes, the AS03-adjuvanted formulations showed a superior immune response. O3b responses were reduced compared to the other vaccine antigens.

**Conclusions:**

Kleb4V is the first *K. pneumoniae* conjugate vaccine candidate to reach clinical assessment. The Kleb4V bioconjugate was immunogenic and well tolerated in the target population of adults aged 55–70 years for the vaccine-serotypes.

## Teaser

Safe and effective vaccines are urgently needed to prevent infections caused by *Klebsiella pneumoniae*. This first-in-human study demonstrates that a vaccine containing 4 *K. pneumoniae*–derived O antigens is safe and induces both binding and functional antibodies.

*Klebsiella pneumoniae* is a major cause of infections in humans [1]. In high-income countries, the burden of *K. pneumoniae* is driven primarily by healthcare-associated infections, accounting for 6%–17% of healthcare-associated infections, among vulnerable patients with comorbid conditions or invasive devices (eg, catheters or mechanical ventilation) [2]. The burden of disease due to *K. pneumoniae* is higher in low- and middle-income countries, where limited healthcare infrastructure contributes to an increased incidence of both hospital-acquired and community-acquired infections [2]. In these countries, *K. pneumoniae* is a substantial contributor to the deaths of infants and neonates in the first 2 years of life [3]. In 2019, it was the second leading pathogen in deaths attributed to antimicrobial-resistant bacteria, particularly in South Asia, sub-Saharan Africa, and parts of Eastern Europe [4, 5].

In recent decades, the increase in the acquisition of resistance to a wide range of antibiotics among *K. pneumoniae* strains has severely limited treatment options. This growing public threat has prompted the World Health Organization to classify *K. pneumoniae* as a priority 1 pathogen, prioritizing the development of treatment strategies and vaccines [6].

There have been numerous efforts to develop vaccines against *K. pneumoniae* [7], including the use of attenuated bacteria, inactivated whole cells, polysaccharides (PSs) and lipopolysaccharides, proteins, and ribosomal vaccines [8–14], but no licensed vaccines are currently available. The success of glycoconjugate vaccine for other encapsulated bacteria has increased interest in a similar approach for *Klebsiella* vaccine development.

*K. pneumoniae* produces 2 key surface PSs that play an essential role in its virulence. Capsular PS (CPS) designated as the *K antigen*, consists of a repeating sugar matrix structure covering the outer bacterial layer, while a key structural element of the outer component of the gram-negative outer membrane is lipopolysaccharide, a conserved lipid A region and an external O-antigen PS [15]. Eleven unique serotypes have been identified for the O antigen, compared with >100 different K structures. A vaccine targeting the O antigen has been proposed as a rational approach, considering the limited number of O structures [15–17]. Epidemiological studies showed that O1, O2, O3, O4, and O5 are the top prominent O types responsible for >80% of infections in Africa and South Asia, regions that bear the greatest burden of *K. pneumoniae antibiotic* resistance [18, 19].

In view of this, LimmaTech Biologics has developed a *K. pneumoniae* tetravalent bioconjugate vaccine (Kleb4V) comprising the O1v1, O2a, O2agf, and O3b serotypes linked to the detoxified recombinant exoprotein A of *Pseudomonas aeruginosa* (EPA). The use of EPA aims to induce immunologic memory and enhance immunogenicity to the PS. According to the available data and assuming cross-protection between the O1 serotypes (O1v1 and O1v2), Kleb4V is projected to provide coverage for approximately 70% of *K. pneumoniae* strains causing human infection worldwide, although this may vary by region. Here we describe a first-in-human phase I trial designed to assess the safety and immunogenicity of this vaccine.

## METHODS

### Study Design and Participants

This was a first-in-human phase I/II, observer-blind, randomized, placebo-controlled study to evaluate the safety and immunogenicity of 2 doses of a candidate *K. pneumoniae* bioconjugate vaccine (Kleb4V), administered intramuscularly twice, 2 months apart, with or without AS03 (a proprietary adjuvant system of GSK). The study was conducted in 2 steps (Supplementary Figure 1). In step 1 (safety cohort), 16 healthy adults 18–40 years old were enrolled in a staggered fashion in 2 groups (groups 1 and 2; 8 per group) and sequentially administered the Kleb4V target dose (64 µg), nonadjuvanted and thereafter adjuvanted with AS03, or placebo (phosphate-buffered saline). After this, 32 healthy adults 55–70 years old were enrolled via a staggered dose escalating (16 and 64 µg) approach (groups 3–6; 8 per group), with the group receiving the nonadjuvanted dose enrolled before the group receiving the adjuvanted one. In step 2 (target cohort), 118 healthy adults 55–70 years old were concomitantly randomized to any Kleb4V dose (with or without AS03) or placebo. Approval was obtained from the Ethics Committee of the Bavarian State Chamber of Physicians in Munich, and all participants provided written informed consent. The eligibility criteria, demographic data, and other baseline characteristics of the participants are included in Supplementary Tables 1 and 2.

### Study Vaccine

Kleb4V contained O-antigen PSs of *K. pneumoniae* serotypes O1v1, O2a, O2afg, and O3b, each conjugated to the EPA. The target dose contained 16 µg of glycan per serotype, for a total of 64 µg of PS, and the low dose contained 4 µg of glycan per serotype, for a total of 16 µg of PS. The trial was conducted in 2 clinical sites in Germany (Nuvisan sites in Neu Ulm and Gauting).

### Randomization and Masking

The study was observer blind. Healthy volunteers, sites personnel, Data and Safety Monitoring Committee, sponsor and Clinical Research Organisation study teams were blinded, except for designated representatives (including the pharmacist, the assigned unblinded biostatistician, and the monitor). The randomization list was generated by the CRO and kept in a safe location with restricted access. Blinding was maintained throughout the study. In the first 2 groups of step 1, two sentinels were randomized 1:1; and 6 additional participants per group were randomized 5:1. The randomization was 3:1 in groups 3–6 of step 1. Participants in step 2 were randomized to receive Kleb4V in a low (16-µg) or target (64-µg) dose, both with or without AS03 (n = 24 each), or placebo (n = 22).

### Procedures

Study participants had 8 onsite visits in step 1 and 9 in step 2, including screening, 2 vaccination visits, follow-up visits 7 days after each injection, 14 days after the first injection (step 2 only), 1 month after the first and second injections, and finally 6 months after the last injection (Supplementary Figure 2).

At each vaccination a diary card was provided for the participant to collect solicited and unsolicited adverse events (AEs) in the 7 days following each vaccination. Unsolicited AEs were collected for 28 days after each injection. Any serious AEs (SAEs), AEs of special interest (ie, potential immune-mediated diseases and any AEs leading to vaccine/study withdrawal) were collected for the entire study duration. At screening, vaccination visits, and at the visit 7 days after each vaccination, blood samples were obtained to test safety parameters. Blood samples were also obtained to test the immune response against Kleb4V antigens, on the day of each vaccination, 14 days after the first injection (in step 2 only), 1 month after each vaccination, and at the final follow-up visit.

### Outcomes

The primary objective was to obtain first-in-human safety and immunogenicity data after administration of Kleb4V to 55–70-year-old adults and to identify the preferred formulation of Kleb4V. The safety assessment focused on the occurrence, severity, and relationship of solicited and unsolicited AEs during the 7 and 28 days, respectively, after each vaccination, as well as the occurrence of SAEs and other medically relevant AEs during the entire study duration. The geometric mean titers (GMTs) of serum immunoglobulin (Ig) G against the O serotypes included in Kleb4V, at baseline and 28 days after the second vaccination, were evaluated for the primary immunogenicity assessments.

### Antibody Measurements

Serum samples from vaccinees obtained on days 15, 29, 85, and 225 were assessed. IgG titers against the 4 vaccine serotypes were measured using a qualified multiplexed bead assay developed in the University College London laboratory [20]. Functional immunity to O1v1, and O1v2 (assessing cross-reactive immunity with O1v1) was measured by means of a functional opsonophagocytic killing assay (OPA), optimized and qualified at the University College London, while functional immunity to O2afg was measured using a serum bactericidal assay [20]. No functional assay for O2a or O3b existed when the samples were available for analysis.

### Statistical Analysis

The sample size for the target population (aged 55–70 years) was driven by the primary immunogenicity analyses (GMTs 28 days after the second vaccination) and for each of the 5 treatment arms was estimated to be 30 participants, assuming the following: IgG responses in a log-normal distribution; ≥2.5-fold difference in IgG (geometric mean ratio [GMR], ≥2.5) in each comparison; α = .0125 (2 sided) and β = .1 (ie, 90% power); and an overall drop-out rate of 14% (steps 1 and 2). The sample size of 150 participants was obtained by combining 32 from step 1 and 118 from step 2. For each treatment group except the placebo group, 6 participants came from step 1 and 24 from step 2; for the placebo group, 8 came from step 1 and 22 from step 2.

The immunogenicity end points were evaluated in the per-protocol population of participants who completed the 2-dose vaccination schedule. Immunogenicity objectives included evaluating the GMR, IgG responses expressed as the ratio between the baseline and the postvaccine time point of interest. The GMR was analyzed using analysis of covariance, with treatment as a fixed factor and baseline titer as a covariate. Dunnett's procedure was used to compare the 4 active treatment groups with the placebo group, accounting for multiplicity, with a study-wide α level of .05. For these primary comparisons, treatment ratios, adjusted *P* values and 2-sided confidence intervals (CIs) with adjusted coverage were computed following the back-transformation of the results to the original scale. Also calculated were the number and percentage of vaccinees presenting a ≥4-fold increase in their IgG titers against the 4 serotypes, referred as to as *responders*. The corresponding exploratory 95% confidence intervals were calculated using the Clopper-Pearson method.

A summary of the descriptive statistics for antibody functionality included the minimum, maximum, and median OPA/serum bactericidal assay titers for each serotype across the treatment groups, along with the baseline GMT and GMR, all presented with 95% confidence intervals. The safety end points were evaluated descriptively in the safety population, which comprised participants who received ≥1 dose of the vaccine.

## RESULTS

**Figure 1:**
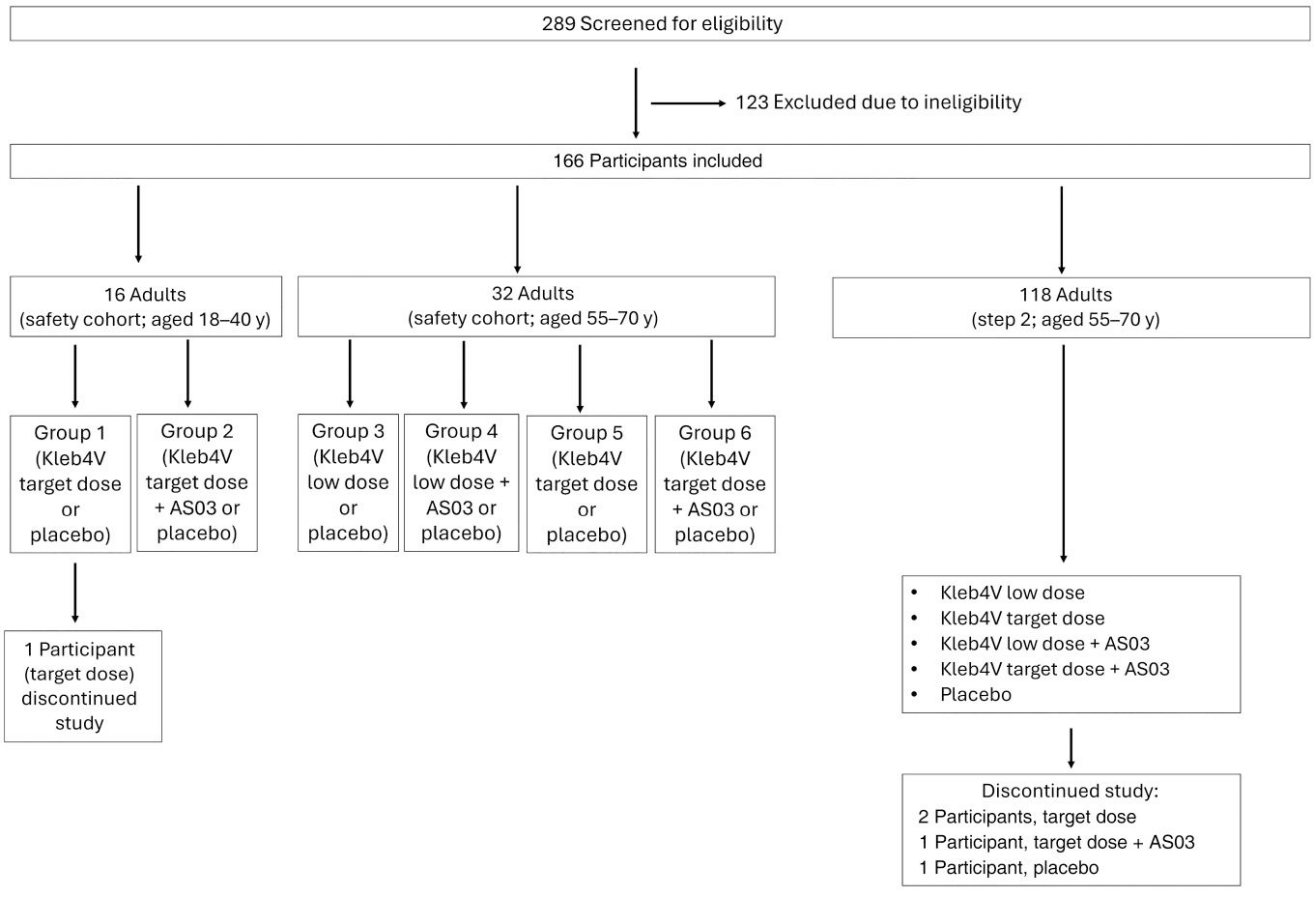
Trial profile. Selection and screening and randomization of patients at the Neu-Ulm and Gauting sites in Germany, where 124 and 42 participants were included, respectively. They were randomized and treated, and 161 completed the study as planned. All study participants were treated with 2 doses of the same formulation—*Klebsiella pneumoniae* tetravalent bioconjugate vaccine (Kleb4V), target dose (64 µg), with or without AS03 adjuvant; Kleb4V), low dose (16 µg), with or without AS03; or placebo—and were included in the safety set. Those who did not receive the second vaccination and/or had no immunogenicity evaluation available for the visit 1 month after the second vaccination (visit 8) were excluded from the immunogenicity analysis set.

Between 29 June 2021 and 26 September 2022, a total of 289 individuals were screened; 166 were included and randomly assigned, and 161 completed the study and were eligible for immunogenic assessment (Figure 1). Five participants (4 vaccinees and 1 placebo recipient) dropped out for reasons unrelated to vaccination or safety events (4 after the first and 1 after the second vaccination). The mean ages (SDs) of participants in the target group who received the different formulations were 60.7 (4.13) years for the low dose, 61.1 (4.36) years for the low adjuvanted dose, 60.2 (4.96) years for the target dose, 61.5 (4.96) years for the target adjuvanted dose and 60.4 (4.00) years for the placebo.

No potential immune-mediated diseases were reported in this study, and most AEs were of mild to moderate intensity (Supplementary Table 3). All participants (n = 12) aged 18–44 years, who received either 64-μg or 64-μg Kleb4V adjuvanted with AS03, reported solicited AEs related to vaccination, primarily localized reactions at the injection site, and 2 reported unsolicited AEs. Among the 4 participants aged 18–44 years administered placebo, 2 reported solicited and unsolicited AEs, which were considered related to the investigational product. In the target age group (55–70 years), the incidence of solicited AEs deemed related to vaccination was higher in the vaccinated groups than in the placebo group, namely 60% (n = 18) and 90.0% (n = 27) in the 16-μg groups without or with AS03, respectively; 80% (n = 24) and 96.7% (n = 29) in the 64-μg groups without or with AS03, respectively; and 36.7% (n = 11) in the placebo group.

For both age groups, there was no difference in the numbers of study participants reporting solicited AEs after the first and second doses, but the severity of AEs increased after the second vaccination. Among the 18–40-year-old participants, AEs of severe intensity occurred in only 2 participants after the second Kleb4V vaccination (for the adjuvanted and not the adjuvanted formulation). In the older group, the numbers of participants with severe solicited AEs were similar after the first and second vaccinations, except in the group receiving 16-µg Kleb4V with AS03, where more participants reported severe solicited AEs after the second vaccination. Severe local reactions were reported among vaccinees after the first and second vaccinations (4 for each), mostly in the adjuvanted 64-µg Kleb4V group. All reactions were transient and resolved with no sequelae.

A higher incidence of unsolicited AEs related to the investigational product was reported after administration of 64-µg than after16-µg Kleb4V (ie, 16.7% after 16-µg Kleb4V with or without AS03 vs 36.7% after 64-µg Kleb4V alone and 46.7% after 64-µg Kleb4V adjuvanted with AS03) (Supplementary Table 3). Overall, 5 participants experienced 6 SAEs: 1 after receiving 64-µg Kleb4V without AS03, 1 after receiving 64-µg Kleb4V with AS03, and 3 after receiving placebo. All SAE were considered not related to the investigational product.

**Figure 2.**
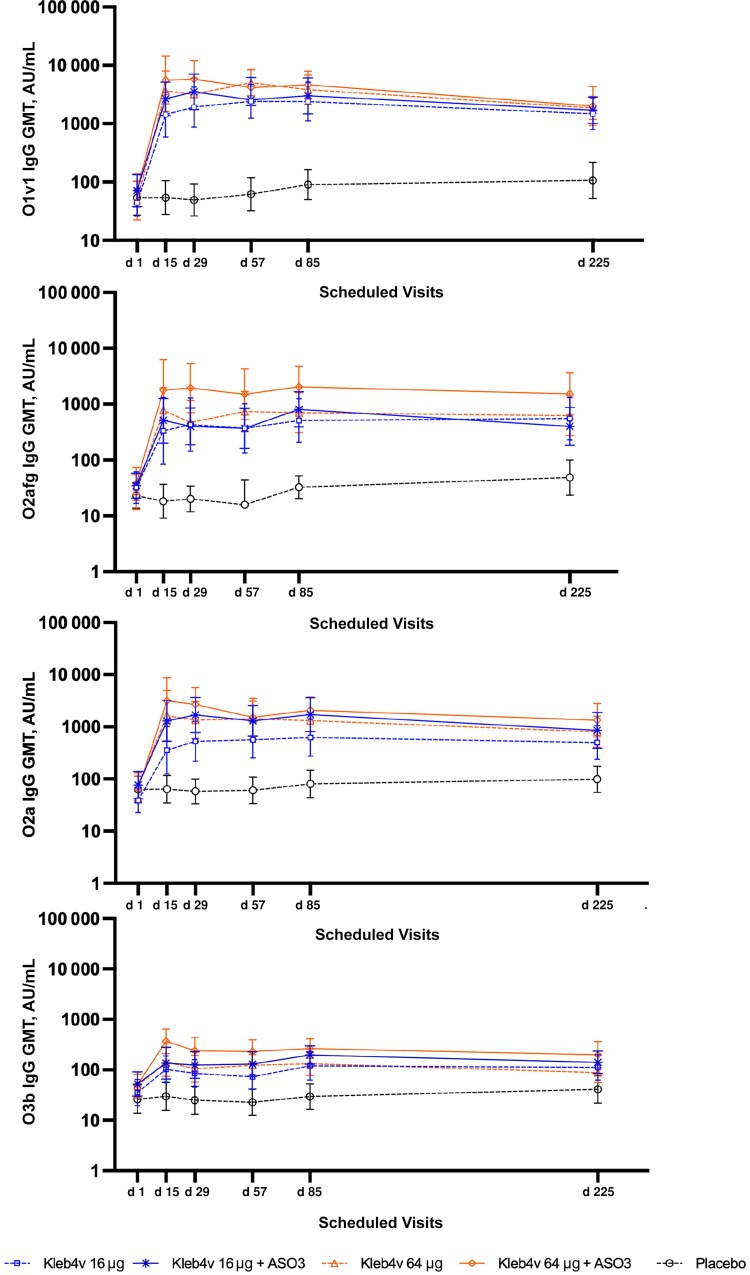
Antibody-specific immunoglobulin (Ig) G to O antigens in serum samples from vaccinees (aged 55–70 years; immunogenicity analysis set), as measured with a multiplex Luminex assay; to O antigens O1v1, O2afg, O2a, and O3b were included in the *Klebsiella pneumoniae* tetravalent bioconjugate vaccine (Kleb4V). Data are expressed as geometric mean titers (GMTs) with 95% confidence intervals. Vaccines were administered on days 1 and 57, and antibody titers were measured at each of the 6 visits on the days indicated. Abbreviation: AU, arbitrary units.

The geometric mean serotype-specific IgG titers (GMTs), 28 days after the second vaccination (day 85) in the target group (participants aged 55–70 years), showed a statistically significant increase for each of the 4 serotypes in all vaccine groups compared with placebo (*P* < .001) (Figure 2).

IgG responses, expressed as the ratio between the baseline and the day 85 time point, are shown in Table 1. In general, the GMR was highest for O1v1 and lowest for O3b (Table 1). The reduced immunogenicity of O3b may be attributable to its distinctive glycan structure, which is rich in mannan, similar to that described for the *Escherichia coli* O8 serotype commonly found in humans and therefore more likely to be tolerated [21]. The Kleb4V 64-µg formulation was in general the most immunogenic and, when adjuvanted with AS03, resulted in improved responses to O2a, O2afg, and O3b (Table 1 and Figure 2). IgG responses on days 15 and 29 following receipt of the first dose can also be seen in Figure 2. While this was not part of the formal analysis, it is clear that in general responses to the 4 O antigens in the vaccine developed rapidly over the first 4 weeks after vaccine receipt , with little increase following the second vaccination.

**Table 1.** Comparison of Serum Immunoglobulin G Titers Specific to O1v1, O2a, O2afg, and O3b Between Baseline and Day 85 (Visit 8) in the Target Population (Study Participants Aged 55–70 Years)

Vaccine Group	No.	Serotype O1v1				Serotype O2a				Serotype O2afg				Serotype O3b			
		GMT (95% CI)	GMR	*P* Value^a^	≥4-Fold Increase, No. (%; 95% CI)	GMT (95% CI)	GMR	P Value^a^	≥4-Fold Increase, No. (%; 95% CI)	GMT (95% CI)	GMR	*P*Value^a^	≥4-Fold Increase, No. (%; 95% CI)	GMT (95% CI)	GMR	*P* Value^a^	≥4-Fold Increase, No. (%; 95% CI)
Kleb4V, 16 µg	29	2709.3 (1582.2–4639.4)	29.4	<.001	27 (93.1; 77.2–99.2)	853.3 (483.2– 1507.1)	10.8	<.001	24 (82.8; 64.2–94.2)	487 (273.2–868.0)	12.1	<.001	21 (72.4; 52.8–87.3)	126.9 (81.5– 197.7)	3.5	<.001	12 (41.4; 23.5–61.1)
Kleb4V , 16 µg + AS03	30	2520.4 (1472.3– 4314.7)	27.3	<.001	25 (83.3; 65.3–94.4)	1398.1 (794.9–2458.8)	17.8	<.001	22 (73.3; 54.1–87.7)	690.4 (387.4– 1230.7)	17.2	<.001	26 (86.7; 69.3–96.2)	168.8 (108.4–263.0)	4.7	<.001	16 (53.3; 34.3–71.7)
Kleb4V, 64 µg	29	4115.5 (2405.0–7042.6)	44.6	<.001	28 (98.6; 82.2–99.9)	1161.6 (660.4–2043.2)	14.8	<.001	24 (82.8; 64.2–94.2)	757.1 (424.7– 1349.6)	18.9	<.001	25 (86.2; 68.3–96.1)	121.6 (78.1– 189.5)	3.4	<.001	10 (34.5; 17.9–54.3)
Kleb4V, 64 µg + AS03	29	4107.5 (2398.8–7033.4)	44.5	<.001	27 (93.1; 77.2–99.2)	1984.6 (1129.1–3488.4)	25.2	<.001	26 (89.7; 72.6–97.8)	1692.6 (948.5–3020.5)	42.2	<.001	27 (93.1; 77.2–99.2)	238.6 (153.1–371.9)	6.6	<.001	15 (51.7; 32.5–70.6)
Placebo	29	92.3 (54.0– 157.9)	…	…	4 (13.8; 3.9–31.7)	78.7 (44.7– 138.2)	…	…	1 (3.4; 72.6–97.8)	40.1 (22.5–71.6)	…	…	5 (17.2; 5.8–35.8)	35.9 (23.0–56.2)	…	…	5 (17.2; 5.8–35.8)

Abbreviations: CI, confidence interval; GMR, geometric mean ratio (active vs placebo); GMT, geometric mean titer; Kleb4V, *Klebsiella pneumoniae* tetravalent bioconjugate vaccine.

^a^Dunnett *P* values.

The percentage of participants with ≥4-fold IgG responses on day 85 compared with baseline were high for the adjuvanted 64-µg formulation, and in a similar range for serotypes O1v1, O2a, and O2afg (range, 89%–93%; Table 1). Percentages were lower for O3b, although increased for the recipients of the 2 adjuvanted formulations (approximately 50%).

At the final visit 6 months after the second vaccination (day 225), most of the target population, administered with the adjuvanted 64-µg formulation, still showed GMTs ≥4-fold higher than at baseline for O1v1 (96%), O2a, and O2afg (89%) (Supplementary Table 4), and about half of them (48%) for serotype O3b.

**Figure 3.**
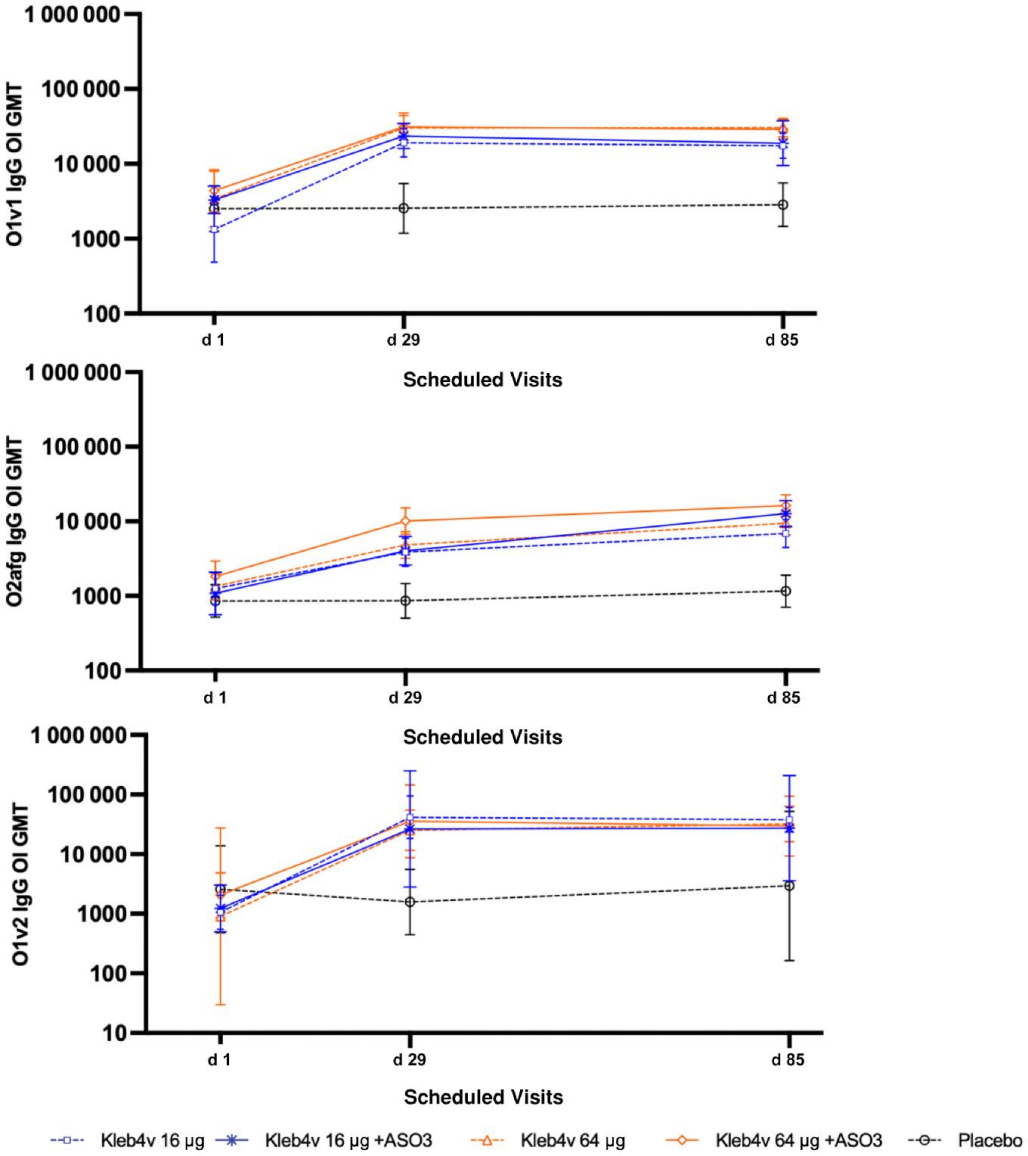
Functional activity of antibody-specific immunoglobulin (Ig) G in serum samples from vaccinees (aged 55–70 years; immunogenicity analysis set) specific to the O antigens O1v1, O2afg, and O1v2. The O1v1 and O2afg antigens were included in the vaccine, and O1v2 was tested for cross-reactivity. Functional activity was measured at each visit using an opsonophagocytic (O1v1 and O1v2) or a bactericidal (O2afg) assay and expressed in geometric mean titers (GMTs) of opsonic indices (OIs) and 95% confidence intervals. Vaccines were administered on days 1 and 57, and antibody titers were measured 28 days after the first and second vaccinations.

The functionality of the antibody response generated after vaccination was evaluated for the vaccine serotypes O1v1 and O2afg by means of the OPA and a serum bactericidal assay, respectively. These were the only 2 assays available and qualified when the study was undertaken. There was a significant increase over and above baseline in functional antibody for all vaccine groups on day 85 (Figure 3 and Table 2) although functionality did not seem correlated with the amount of antigen or the presence of adjuvant. For O1v1, the fold increases above baseline measured on day 29 were slightly higher than those on day 85, with no clear impact of AS03. For O2afg, fold increases rose between day 29 and day 85, with the greatest increases in the group who received the unadjuvanted 64-μg formulation (44.8% increased to 58.6%). Cross-reactivity toward serotype O1v2, not included in the vaccine, was also measured with OPA. The overall pattern of functional antibody after vaccination mirrored that of O1v1 (Figure 3), although fold increases over baseline were significantly lower than those seen for O1v1 (Table 2) on both day 29 and day 85. There was a clear correlation between antibody titers and functional activity for O1v1 (*r* = 0.77; *P* < .001) and O2afg (*r* = 0.55; *P* < .001) (Supplementary Figure 3).

**Table 2.** Summary Data From Opsonophagocytic Assay (O1v1 and O1v2) and Serum Bactericidal Assay (O2afg) for the Different O Serotypes in the Baseline Population (Aged 55–70 Years)

Serotype	Visit No.	≥4-Fold Increase in GMT by Treatment Group, No. (%; 95% CI)				
		Kleb4V, 16 µg (n = 29)	Kleb4V, 16 µg + AS03 (n = 30)	Kleb4V, 64 µg (n = 29)	Kleb4V, 64 µg + AS03 (n = 29)	Placebo (n = 29)
O1v1 (OPA)	5 (d 29)	16 (55.2; 35.7–73.6)	15 (50.0; 31.3–68.7)	18 (62.1; 42.3–79.3)	15 (51.7; 32.5–70.6)	0 (0.0)
	8 (d 85)	17 (58.6; 38.9–76.5)	14 (46.7; 28.3–65.7)	17 (58.6; 38.9–76.5)	12 (41.4; 23.5–61.1)	1 (3.4; 0.1–17.8)
O1v2 (OPA)	5 (d 29)	6 (20.7; 54.1–100)	3 (10.0; 11.8–88.2)	3 (10.3; 11.8–88.2)	5 (17.2; 35.9–99.6)	0 (0.0)
	8 (d 85)	6 (20.7; 54.1–100)	4 (13.3; 22.3–95.7)	4 (13.8; 22.3–95.7)	6 (20.7; 54.1–100)	0 (0.0)
O2afg (SBA)	5 (d 29)	10 (34.5; 17.9–54.3)	7 (23.3; 9.9–42.3)	14 (48.3; 29.4–67.5)	13 (44.8; 26.4–64.3)	0 (0.0)
	8 (d 85)	15 (51.7; 32.5–70.6)	19 (63.3. 43.9–80.1)	21 (72.4; (52.8–87.3)	17 (58.6; 38.9–76.5)	3 (10.3; 2.2–27.4)

Abbreviations: CI, confidence interval; Kleb4V, *Klebsiella pneumoniae* tetravalent bioconjugate vaccine; OPA, opsonophagocytic assay; SBA, serum bactericidal assay.

## DISCUSSION

This is the first-in-human study of a novel *Klebsiella* bioconjugate vaccine. All Kleb4V formulations were generally well tolerated; 8 participants experienced severe local reactions (6% of all receiving Kleb4V), 6 in the groups receiving the AS03-adjuvanted vaccine formulations. AEs were limited and resolved without any lasting effects. The majority of both solicited and unsolicited AEs were mildly to moderately severe. The Kleb4V bioconjugate was shown to be immunogenic, with a high percentage of study participants exhibiting a ≥4-fold increase in IgG against O1v1, O2a, and O2afg at all visits, compared with baseline. In general, the 64-μg formulations were superior to the 16-g ones, and the adjuvanted formulations triggered a better immune response than the nonadjuvanted ones. Such an effect was less evident for serotype O1v1, probably because of its generally higher immunogenicity compared with the other vaccine serotypes.

Multiple vaccine modalities have been explored for *K. pneumoniae* over the past 5 decades. Early whole-cell or lysate preparations demonstrated immunogenicity in small studies but raised safety and standardization concerns [10, 12, 22, 23]. Subsequent efforts focused on vaccine development based on CPS. A notable historical effort was a 24-valent CPS vaccine (including K2, K3, K5, K9, K10, K15–18, K21, K22, K25, K28, K30, K35, K43, K52, K53, K55, and K60–64) that entered a phase 1 clinical trial and was immunogenic and well tolerated when coadministered with a *Pseudomonas* O-antigen conjugate [9, 24, 25]. However, CPS formulations face inherent challenges, with >100 000 serotypes, substantial geographic variability, and the poor immunogenicity of unconjugated PS in young children [26].

Therefore, efforts have included a focus on O-antigen–based conjugate strategies with ≥11 O serotypes being recognized, compared with >100 capsular K antigens [18, 27]. In particular, serotypes O1, O2, O3, O4, and O5 typically account for approximately 80%–90% of clinical isolates, supporting the rationale for multivalent O-antigen vaccines. Encouraging preclinical data were provided in 2018 by Hegerle et al [28], who reported a quadrivalent O-antigen conjugate (O1, O2, O3, and O5) linked to *Pseudomonas* flagellin that elicited functional antibodies to the 4 O-antigen PS types and protection in mice and rabbit models.

Similarly, a heptavalent O-antigen bioconjugate vaccine (Omniose; O1v1, O1v2, O2a, O2afg, O3, O3b, and O5) was developed by Wantuch and colleagues [11], who reported their preclinical evaluation. This vaccine showed immunogenicity in animals against each of the 7 bioconjugates, with limited cross-reactivity among subtypes and variable complement-mediated killing across strains. In findings similar to ours in humans, O1v1 IgG rabbit antiserum recognized both O1v1 and O1v2 bacterial serotypes [11].

These immunogenicity results underscore the promise of O-antigen targeting and suggest that epitope accessibility is possible with an O-antigen vaccine. This is relevant as several studies have suggested that the capsule may sterically mask underlying O antigens, reducing the accessibility of anti-O antibodies and potentially limiting functional efficacy [29, 30]. Therefore, in designing Kleb4V, we focused on the O1v1, O2a, O2afg, and O3b antigens because they are more conserved across clinically relevant isolates, are accessible to antibodies on the bacterial surface, and have demonstrated functional bactericidal activity in preclinical models.

The less prevalent, though regionally significant, serotype O5 was not included in Kleb4V. Similar to serotype O3, O5 consists of a linear polymannose chain. However, despite the comparable mannose linkages, studies report limited cross-reactivity between these serotypes, likely due to the β-linked mannopyranosyl unique to the O5 repeat unit, which may alter the overall O-antigen conformation and immunogenicity [27]. Inclusion of the O5 serotype in future generation of multivalent *Klebsiella* vaccines could therefore be considered. While anti-CPS immunity is likely to be important for protective immunity, O-antigen–targeted strategies, if successful, will provide broader strain coverage with fewer vaccine antigens.

Two recent efforts toward a *Klebsiella* conjugate vaccine are under early development, with no publicly available data, to our knowledge. A conjugate vaccine is being designed at the University of Maryland, to be given to pregnant women from low- and middle-income countries so that they can pass protection to their newborns via maternal antibodies [31]. Another vaccine candidate (CladeVax) is a nasal spray vaccine against MDR *K. pneumoniae* being developed at Tulane University. CladeVax is designed to protect the immunocompromised and the elderly.

In conclusion, Kleb4V has an acceptable safety profile and was immunogenic in healthy adults aged 55–70 years. This target age group was chosen for this phase I/II study to reflect the population most commonly exposed to *Klebsiella* infections, especially in nosocomial environments.

The absence of an immunocorrelate or surrogate of protection currently represents a limitation and means that an efficacy trial of a *Klebsiella* vaccine with a clinical end point would be required. However, the extensive sample size required for such an efficacy trial remains a concern, despite recent efforts to identify populations at elevated risk of *Klebsiella* infection in order to de-risk a clinical proof-of-concept efficacy study [32]. Should an immune correlation become available (for example from epidemiological studies with mothers and newborns), the data available from Kleb4V could be potentially useful for predicting clinical efficacy. To date, to our knowledge, Kleb4V is the only *Klebsiella* bioconjugate vaccine tested in phase I/II trials in humans, and its positive safety and immunogenicity outcomes pave the way to further its clinical development, expanding on formulation studies and efficacy clinical proof-of-concept trials.

## Supplementary Material

jiaf600_Supplementary_Data

## Supplementary Data

Supplementary materials are available at *The Journal of Infectious Diseases* online (http://jid.oxfordjournals.org/). Supplementary materials consist of data provided by the author that are published to benefit the reader. The posted materials are not copyedited. The contents of all supplementary data are the sole responsibility of the authors. Questions or messages regarding errors should be addressed to the author.
