# Supplementary material for: Safety and Immunogenicity of a Klebsiella pneumoniae Tetravalent Bioconjugate Vaccine (Kleb4V) Administered to Healthy Adults: A First-in-Human Phase I/II Randomized and Controlled Study
Source: J Infect Dis. 2025 Nov 25;233(2):e342–51. doi: 10.1093/infdis/jiaf600 (PMC13017035; doi:10.1093/infdis/jiaf600)
Supplement: jiaf600_Supplementary_Data [file jiaf600_supplementary_data.docx]

**Supplementary material**


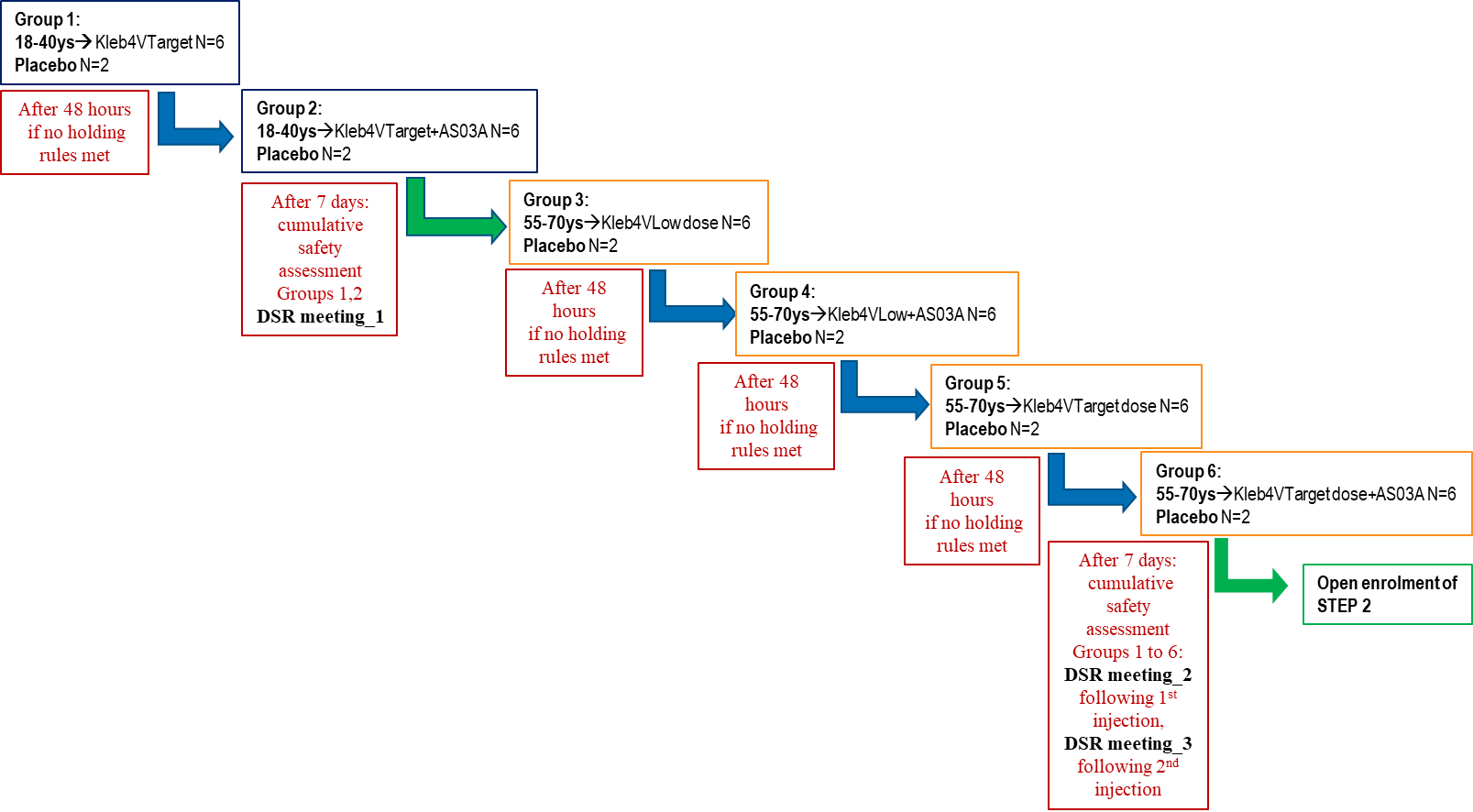


Figure S1: Two-step study enrolment chart. Step 1 (safety cohort) involved staggered enrolment, beginning with a small group of young adults (18–40 years) who sequentially received the Kleb4V target dose with or without AS03, or placebo. This was followed by older adults (55–70 years) who received the Kleb4V low dose with or without AS03, and subsequently the Kleb4V target dose with or without AS03, or placebo. Step 2 (target cohort) included adults aged 55–70 years randomized to receive either the Kleb4V low dose with or without AS03, the Kleb4V target dose with or without AS03, or placebo.

**Screening**

**V1**

**Visit 2**

Day 1

**Visit 3**

Day 8

**Visit 4**

Day 15

Day 58

**Phone Call 1**

Day 2

**Visit 5**

Day 29

**Visit 7**

Day 64

Diary Completion

**Visit 6**

Day 57

**Phone Call 3**

Day 155

**Phone Call 2**

**Visit 8**

Day 85

**Visit 9**

Day 225

Diary Completion

Figure S2: Schedule of step 2 in study visits. The participants completed nine onsite visits, also supplemented by three interim phone calls.

Figure S3. Correlation between binding and functional activities of O-Klebsiella antibodies (O1v1; r=0.77) and (O2afg; r=0.55), *p*<0.0001.

Table S1. List of Eligibility Criteria: Inclusion and Exclusion

| **Inclusion criteria** |
| --- |
| 1. Good general health by medical history, laboratory findings and physical examination before receiving vaccination as judged by the investigator (subjects with a minor controlled illness, such as mild controlled hypertension, asthma or COPD, and without fever might be enrolled at the discretion of the investigator) |
| 1. Subject who was willing and able to comply with the requirements of the protocol (e.g. completion of the diary cards, return for follow-up visits) |
| 1. Signed written informed consent obtained from the subject |
| 1. For Step 1 Groups 1 and 2 only: Female or male between 18-40 years (inclusive) of age at the time of the 1^st^ vaccination |
| 1. For Step 1 Groups 3 to 6, and Step 2: Female or male subjects between 55-70 (inclusive) years of age at the time of 1^st^ vaccination |
| 1. Female subjects of childbearing potential were eligible, as long as they had practiced adequate contraceptive measures from two months before the 1^st^ vaccination until one month after the last vaccination. |
| **Exclusion criteria** |
| 1. Health condition that, in the opinion of the investigator, might interfere with optimal participation in the study or place the volunteer at increased risk of AEs. Study clinicians, in consultation with the principal investigator, used clinical judgment on a case-by-case basis to assess safety risks under this criterion |
| 1. Any clinically significant deviation from the normal range in biochemistry or haematology blood tests in the opinion of the investigator |
| 1. Clinically significant abnormalities on physical examination |
| 1. Suspected or known hypersensitivity (including allergy) to any of the vaccine components or to medicinal products or medical equipment whose use is foreseen in this study |
| 1. History of allergy to any vaccine |
| 1. Clinical conditions representing a contraindication to intramuscular vaccination and blood draws |
| 1. Acute or chronic, clinically significant cardiovascular, pulmonary, hepatic or renal abnormality diseases and/or insufficiency as determined by physical examination or laboratory tests. |
| 1. Known or suspected impairment of immunological function, documented Human Immunodeficiency Virus (HIV) infection, asplenia/splenectomy, or history of autoimmune disease or lymphoprolipherative disorder |
| 1. Positive blood test for HBsAg, HCV, HIV-1/2 2. Positive test for SARS-CoV-2 |
| 1. History of systemic administration of immunosuppressive drugs |
| 1. Administration of antineoplastic and immune-modulating agents or chemotherapy within 90 days prior to informed consent |
| 1. Planned administration of a vaccine not foreseen by the study protocol within 4 weeks prior to 1^st^ vaccination and 4 weeks after last vaccination. Vaccination against seasonal influenza virus (or Covid vaccine) was allowed outside of +/- 7 days from each vaccination |
| 1. Concurrently participating in another clinical study, at any time during the study period, in which the subject had been or was to be exposed to an investigational or a non-investigational interventional vaccine/product (pharmaceutical product) |
| 1. Body Mass Index (BMI) <19 and >30 |
| 1. History of any chronic or progressive disease that according to judgment of the investigator could interfere with the study outcomes or pose a threat to the participant's health |
| 1. Received an investigational or non-registered product (medicinal drug or vaccine), other than the study vaccine within 3 months prior to 1^st^ administration of study vaccine, or planned use during the study period |
| 1. Administration of immunoglobulin and/or any blood products within the 3 months preceding the first dose of study vaccine |
| 1. Blood donation of at least 500 mL blood draw within 3 months preceding injection or planned during the study period as reported by subject |
| 1. Use of any antibiotic therapy within 1 week preceding each injection |
| 1. Subjects with an elective surgical intervention, planned during the study period until 30 days after 2^nd^ vaccination |
| 1. Females lactating, or pregnancy or intention to become pregnant as reported by subject |
| 1. Current and/or history of chronic alcohol consumption and/or drug abuse |
| 1. History of immune-mediated disease |
| 1. Heavy smokers (> 20 cigarettes per day) |

Table S2: Demographic data and other baseline characteristics of the participants.

|  |  | **18-40yo** | | | **55-70yo** | | | | |
| --- | --- | --- | --- | --- | --- | --- | --- | --- | --- |
| **Parameter (Unit)** |  | Kleb4V 64µg (N=6) | Kleb4V 64µg + AS03 (N=6) | Placebo (N=4) | Kleb4V 16µg (N=30) | Kleb4V 16µg + AS03 (N=30) | Kleb4V 64µg (N=30) | Kleb4V 64µg + AS03 (N=30) | Placebo (N=30) |
| **Age (years)** | Mean±SD | 30.0 ± 8.37 | 27.0 ± 4.05 | 30.5 ± 1.91 | 60.7 ± 4.13  55; 70 | 61.1 ± 4.36  55; 70 | 60.2 ± 4.14  55; 68 | 61.5 ± 4.96  55; 70 | 60.4 ± 4.00  55; 69 |
|  | Min-Max | 20; 40 | 22; 33 | 28; 32 |  |  |  |  |  |
| **Height (cm)** | Mean±SD | 174.2 ± 7.31 | 174.5 ± 11.29 | 177.3 ± 10.37 | 168.5 ± 11.22  149; 193 | 174.9 ± 9.15  157; 190 | 172.8 ± 10.40  152; 192 | 173.0 ± 10.36  155; 189 | 175.1 ± 8.35  160; 187 |
|  | Min-Max | 161; 181 | 164; 191 | 164; 186 |  |  |  |  |  |
| **Weight (kg)** | Mean±SD | 69.57 ± 13.138 | 71.25 ± 13.383 | 78.93 ± 7.467 | 73.25 ± 14.185  51.8; 111.0 | 78.75 ± 14.226  50.0; 106.0 | 77.29 ± 10.970  56.4; 105.4 | 75.57 ± 11.066  54.3; 104.6 | 79.24 ± 12.780  52.9; 102.3 |
|  | Min-Max | 53.1; 87.1 | 60.7; 96.8 | 67.9; 83.9 |  |  |  |  |  |
| **BMI (kg/m²)** | Mean±SD | 22.78 ± 2.951 | 23.27 ± 2.420 | 25.15 ± 1.852 | 25.62 ± 2.683  20.3; 29.8 | 25.59 ± 3.166  20.2; 29.9 | 25.85 ± 2.514  20.8; 29.9 | 25.25 ± 2.926  20.2; 30.0 | 25.69 ± 2.592  19.4; 29.9 |
|  | Min-Max | 19.0; 26.9 | 21.0; 26.5 | 23.4; 27.7 |  |  |  |  |  |

Table S3. Solicited and unsolicited adverse events related to the investigational product

**a)**

| **18-40 years** | | **Treatment** | | |  | |
| --- | --- | --- | --- | --- | --- | --- |
| **MedDRA System Organ Class** | **Preferred Term** | **Kleb4V 64µg (N=6) n (%) e** | **Kleb4V 64µg + AS03 (N=6) n (%) e** | **Placebo (N=4) n (%) e** | **Overall (N=16) n (%) e** | |
| **Overall Summary Data Solicited AEs** | **Summary Data** | **6 (100.0) 31** | **6 (100.0) 32** | **2 (50.0) 4** | **14 (87.5) 67** | |
| General disorders and administration site conditions | Summary Data | 6 (100.0) 24 | 6 (100.0) 23 | 2 (50.0) 2 | 14 (87.5) 49 | |
|  | Fatigue | 5 (83.3) 10 | 3 (50.0) 4 | 1 (25.0) 1 | 9 (56.3) 15 | |
|  | Injection site erythema | 2 (33.3) 2 | 2 (33.3) 2 | - | 4 (25.0) 4 | |
|  | Injection site induration | 2 (33.3) 3 | 3 (50.0) 4 | - | 5 (31.3) 7 | |
|  | Injection site pain | 5 (83.3) 8 | 6 (100.0) 11 | 1 (25.0) 1 | 12 (75.0) 20 | |
|  | Injection site swelling | 1 (16.7) 1 | 2 (33.3) 2 | - | 3 (18.8) 3 | |
| Musculoskeletal and connective tissue disorders | Summary Data | 3 (50.0) 3 | 4 (66.7) 5 | 1 (25.0) 1 | 8 (50.0) 9 | |
|  | Myalgia | 3 (50.0) 3 | 4 (66.7) 5 | 1 (25.0) 1 | 8 (50.0) 9 | |
| Nervous system disorders | Summary Data | 3 (50.0) 4 | 2 (33.3) 4 | 1 (25.0) 1 | 6 (37.5) 9 | |
|  | Headache | 3 (50.0) 4 | 2 (33.3) 4 | 1 (25.0) 1 | 6 (37.5) 9 | |
| **Overall Summary Data Unsolicited AEs** | | **Summary Data** | **2 (33.3) 6** | **2 (33.3) 4** | **2 (50.0) 2** | **6 (37.5) 12** |
| Blood and lymphatic system disorders | | Summary Data | 1 (16.7) 1 | 1 (16.7) 1 | - | 2 (12.5) 2 |
|  | | Lymphadenopathy | 1 (16.7) 1 | 1 (16.7) 1 | - | 2 (12.5) 2 |
| Gastrointestinal disorders | | Summary Data | 1 (16.7) 1 | - | - | 1 (6.3) 1 |
|  | | Nausea | 1 (16.7) 1 | - | - | 1 (6.3) 1 |
| General disorders and administration site conditions | | Summary Data | 1 (16.7) 1 | 2 (33.3) 2 | - | 3 (18.8) 3 |
|  |  | Fatigue | - | 1 (16.7) 1 | - | 1 (6.3) 1 |
|  | | Injection site macule | - | 1 (16.7) 1 | - | 1 (6.3) 1 |
|  | | Injection site reaction | 1 (16.7) 1 | - | - | 1 (6.3) 1 |
| Infections and infestations | | Summary Data | 1 (16.7) 3 | - | - | 1 (6.3) 3 |
|  | | Cystitis | 1 (16.7) 2 | - | - | 1 (6.3) 2 |
|  | | Nasopharyngitis | 1 (16.7) 1 | - | - | 1 (6.3) 1 |
| Nervous system disorders | | Summary Data | - | 1 (16.7) 1 | 1 (25.0) 1 | 2 (12.5) 2 |
|  | | Paraesthesia | - | 1 (16.7) 1 | - | 1 (6.3) 1 |
|  | | Presyncope | - | - | 1 (25.0) 1 | 1 (6.3) 1 |
| Skin and subcutaneous tissue disorders | | Summary Data | - | - | 1 (25.0) 1 | 1 (6.3) 1 |
|  | | Rash maculo-papular | - | - | 1 (25.0) 1 | 1 (6.3) 1 |

b)

| **55-70 years** | | | **Treatment** | | | | | |  | | |
| --- | --- | --- | --- | --- | --- | --- | --- | --- | --- | --- | --- |
| **MedDRA System Organ Class** | **Preferred Term** | | **Kleb4V 16µg (N=30) n (%) e** | **Kleb4V 16µg + AS03 (N=30) n (%) e** | **Kleb4V 64µg (N=30) n (%) e** | **Kleb4V 64µg + AS03 (N=30) n (%) e** | **Placebo (N=30) n (%) e** | | **Overall (N=150) n (%) e** | | |
| **Overall Summary Data Solicited AEs** | **Summary Data** | | **18 (60.0) 47** | **27 (90.0) 118** | **24 (80.0) 90** | **29 (96.7) 136** | **11 (36.7) 29** | | **109 (72.7) 420** | | |
| General disorders and administration site conditions | Summary Data | | 18 (60.0) 40 | 27 (90.0) 90 | 24 (80.0) 64 | 29 (96.7) 112 | 9 (30.0) 20 | | 107 (71.3) 326 | | |
|  | Fatigue | | 7 (23.3) 10 | 15 (50.0) 20 | 9 (30.0) 13 | 7 (23.3) 12 | 8 (26.7) 15 | | 46 (30.7) 70 | | |
|  | Injection site erythema | | 4 (13.3) 5 | 7 (23.3) 10 | 6 (20.0) 9 | 9 (30.0) 14 | 1 (3.3) 1 | | 27 (18.0) 39 | | |
|  | Injection site induration | | 3 (10.0) 4 | 5 (16.7) 7 | 5 (16.7) 7 | 9 (30.0) 16 | - | | 22 (14.7) 34 | | |
|  | Injection site pain | | 11 (36.7) 16 | 26 (86.7) 44 | 21 (70.0) 29 | 29 (96.7) 52 | 3 (10.0) 3 | | 90 (60.0) 144 | | |
|  | Injection site swelling | | 4 (13.3) 5 | 6 (20.0) 7 | 3 (10.0) 5 | 13 (43.3) 17 | 1 (3.3) 1 | | 27 (18.0) 35 | | |
|  | Pyrexia | | - | 2 (6.7) 2 | 1 (3.3) 1 | 1 (3.3) 1 | - | | 4 (2.7) 4 | | |
| Musculoskeletal and connective tissue disorders | Summary Data | | 2 (6.7) 2 | 11 (36.7) 14 | 6 (20.0) 9 | 8 (26.7) 10 | 3 (10.0) 3 | | 30 (20.0) 38 | | |
|  | Myalgia | | 2 (6.7) 2 | 11 (36.7) 14 | 6 (20.0) 9 | 8 (26.7) 10 | 3 (10.0) 3 | | 30 (20.0) 38 | | |
| Nervous system disorders | Summary Data | | 4 (13.3) 5 | 10 (33.3) 14 | 8 (26.7) 16 | 10 (33.3) 14 | 3 (10.0) 6 | | 35 (23.3) 55 | | |
|  | Headache | | 4 (13.3) 5 | 10 (33.3) 14 | 8 (26.7) 16 | 10 (33.3) 14 | 3 (10.0) 6 | | 35 (23.3) 55 | | |
| Skin and subcutaneous tissue disorders | Summary Data | | - | - | 1 (3.3) 1 | - | - | | 1 (0.7) 1 | | |
|  | Erythema | | - | - | 1 (3.3) 1 | - | - | | 1 (0.7) 1 | | |
| **Overall Summary Data Unsolicited AEs*** | | | **Summary Data** | **5 (16.7) 13** | **5 (16.7) 13** | **11 (36.7) 27** | **14 (46.7) 40** | | **5 (16.7) 11** | | **40 (26.7) 104** |
| Blood and lymphatic system disorders | | | Summary Data | - | - | 2 (6.7) 2 | 3 (10.0) 3 | | 2 (6.7) 2 | | 7 (4.7) 7 |
|  | | | Leukocytosis | - | - | 1 (3.3) 1 | 1 (3.3) 1 | | 2 (6.7) 2 | | 4 (2.7) 4 |
|  | | | Lymphadenopathy | - | - | 1 (3.3) 1 | 2 (6.7) 2 | | - | | 3 (2.0) 3 |
| Gastrointestinal disorders | | | Summary Data | 2 (6.7) 2 | 1 (3.3) 1 | 3 (10.0) 6 | 4 (13.3) 8 | | - | | 10 (6.7) 17 |
|  | | | Abdominal discomfort | 1 (3.3) 1 | - | - | - | | - | | 1 (0.7) 1 |
|  | | | Abdominal pain | - | - | - | 3 (10.0) 3 | | - | | 3 (2.0) 3 |
|  | | | Aphthous ulcer | - | - | 1 (3.3) 2 | - | | - | | 1 (0.7) 2 |
|  | | | Diarrhoea | - | - | 1 (3.3) 1 | 1 (3.3) 1 | | - | | 2 (1.3) 2 |
|  | | | Dyspepsia | - | - | 1 (3.3) 1 | - | | - | | 1 (0.7) 1 |
|  | | | Irritable bowel syndrome | 1 (3.3) 1 | - | - | - | | - | | 1 (0.7) 1 |
|  | | | Nausea | - | 1 (3.3) 1 | 1 (3.3) 2 | 2 (6.7) 2 | | - | | 4 (2.7) 5 |
|  | | | Tongue dry | - | - | - | 1 (3.3) 1 | | - | | 1 (0.7) 1 |
|  | | | Vomiting | - | - | - | 1 (3.3) 1 | | - | | 1 (0.7) 1 |
| General disorders and administration site conditions | | | Summary Data | 2 (6.7) 5 | 2 (6.7) 2 | 5 (16.7) 6 | 9 (30.0) 20 | | - | | 18 (12.0) 33 |
|  |  |  | Axillary pain | - | - | 1 (3.3) 1 | - | | - | | 1 (0.7) 1 |
|  | | | Chest pain | 1 (3.3) 1 | - | - | - | | - | | 1 (0.7) 1 |
|  | | | Chills | - | - | 1 (3.3) 1 | 1 (3.3) 1 | | - | | 2 (1.3) 2 |
|  | | | Influenza like illness | - | - | - | 1 (3.3) 2 | | - | | 1 (0.7) 2 |
|  | | | Injection site erythema | - | - | 1 (3.3) 1 | 2 (6.7) 2 | | - | | 3 (2.0) 3 |
|  | | | Injection site haematoma | - | 1 (3.3) 1 | 1 (3.3) 1 | 1 (3.3) 1 | | - | | 3 (2.0) 3 |
|  | | | Injection site induration | - | - | - | 1 (3.3) 1 | | - | | 1 (0.7) 1 |
| General disorders and administration site conditions | | | Injection site muscle weakness | - | - | 1 (3.3) 1 | - | | - | | 1 (0.7) 1 |
|  |  |  | Injection site pruritus | 1 (3.3) 3 | 1 (3.3) 1 | 1 (3.3) 1 | 5 (16.7) 8 | | - | | 8 (5.3) 13 |
|  | | | Injection site warmth | - | - | - | 3 (10.0) 3 | | - | | 3 (2.0) 3 |
|  | | | Malaise | - | - | - | 1 (3.3) 1 | | - | | 1 (0.7) 1 |
|  | | | Pain | 1 (3.3) 1 | - | - | - | | - | | 1 (0.7) 1 |
|  | | | Thirst | - | - | - | 1 (3.3) 1 | | - | | 1 (0.7) 1 |
| Infections and infestations | | | Summary Data | 2 (6.7) 3 | 1 (3.3) 1 | 2 (6.7) 2 | - | | 3 (10.0) 4 | | 8 (5.3) 10 |
|  | | | Cystitis | - | - | - | - | | 1 (3.3) 1 | | 1 (0.7) 1 |
|  | | | Ear infection | - | - | - | - | | 1 (3.3) 1 | | 1 (0.7) 1 |
|  | | | Nasopharyngitis | 1 (3.3) 1 | 1 (3.3) 1 | 2 (6.7) 2 | - | | 2 (6.7) 2 | | 6 (4.0) 6 |
|  | | | Rhinitis | 1 (3.3) 2 | - | - | - | | - | | 1 (0.7) 2 |
| Musculoskeletal and connective tissue disorders | | | Summary Data | - | 1 (3.3) 1 | - | 3 (10.0) 4 | | 1 (3.3) 1 | | 5 (3.3) 6 |
|  | | | Arthralgia | - | 1 (3.3) 1 | - | - | | - | | 1 (0.7) 1 |
|  | | | Myalgia | - | - | - | 2 (6.7) 2 | | - | | 2 (1.3) 2 |
|  | | | Pain in extremity | - | - | - | 1 (3.3) 2 | | - | | 1 (0.7) 2 |
|  | | | Periostosis | - | - | - | - | | 1 (3.3) 1 | | 1 (0.7) 1 |
| Nervous system disorders | | | Summary Data | - | 2 (6.7) 2 | 2 (6.7) 4 | 1 (3.3) 1 | | 1 (3.3) 1 | | 6 (4.0) 8 |
|  | | | Disturbance in attention | - | 1 (3.3) 1 | - | - | | - | | 1 (0.7) 1 |
|  | | | Dizziness | - | - | 1 (3.3) 2 | - | | 1 (3.3) 1 | | 2 (1.3) 3 |
|  | | | Hyperaesthesia | - | 1 (3.3) 1 | - | - | | - | | 1 (0.7) 1 |
|  | | | Migraine | - | - | 1 (3.3) 1 | - | | - | | 1 (0.7) 1 |
|  | | | Paraesthesia | - | - | - | 1 (3.3) 1 | | - | | 1 (0.7) 1 |
|  | | | Somnolence | - | - | 1 (3.3) 1 | - | | - | | 1 (0.7) 1 |
| Respiratory, thoracic and mediastinal disorders | | | Summary Data | - | - | 2 (6.7) 2 | 1 (3.3) 1 | | 1 (3.3) 2 | | 4 (2.7) 5 |
|  | | | Cough | - | - | - | - | | 1 (3.3) 2 | | 1 (0.7) 2 |
|  | | | Dyspnoea | - | - | 1 (3.3) 1 | 1 (3.3) 1 | | - | | 2 (1.3) 2 |
|  | | | Oropharyngeal pain | - | - | 1 (3.3) 1 | - | | - | | 1 (0.7) 1 |
| Skin and subcutaneous tissue disorders | | | Summary Data | 1 (3.3) 3 | 1 (3.3) 2 | 2 (6.7) 3 | - | | - | | 4 (2.7) 8 |
|  | | | Angiodermatitis | - | - | 1 (3.3) 1 | - | | - | | 1 (0.7) 1 |
|  | | | Blister | - | - | 1 (3.3) 1 | - | | - | | 1 (0.7) 1 |
|  | | | Eczema | - | 1 (3.3) 2 | - | - | | - | | 1 (0.7) 2 |
|  | | | Erythema | 1 (3.3) 1 | - | - | - | | - | | 1 (0.7) 1 |
|  | | | Pruritus | 1 (3.3) 2 | - | 1 (3.3) 1 | - | | - | | 2 (1.3) 3 |

N= total number of subjects; n=number of subjects having the event, e=number of events (all events considered)

*Only system organ classes with overall more the 2 subjects reporting AEs in this class are displayed

Table S4. Proportion of the two populations studied, (55-70 and 18-40 years (IAS)), with 4-fold increases in serum IgG from baseline for the four serotypes included in the vaccine.

|  | | | **Treatment** | | | | | | | |
| --- | --- | --- | --- | --- | --- | --- | --- | --- | --- | --- |
|  |  |  | **Population 55-70 yr** | | | | | **Population 18-40 yr** | | |
| **Serotype** | **Visit** |  | **Kleb4V 16µg (N=29)** | **Kleb4V 16µg + AS03 (N=30)** | **Kleb4V 64µg (N=29)** | **Kleb4V 64µg + AS03 (N=29)** | **Placebo (N=29)** | **Kleb4V 64µg (N=5)** | **Kleb4V 64µg + AS03 (N=6)** | **Placebo (N=3)** |
| O1v1 | V5 (D29) | n | 26 | 26 | 27 | 27 | 0 | 5 | 6 | 1 |
|  |  | Percentage | 89.7 | 86.7 | 93.1 | 93.1 | 0.0 | 100.0 | 100.0 | 33.3 |
|  |  | 95% CI in %* | (72.6;97.8) | (69.3;96.2) | (77.2;99.2) | (77.2;99.2) | - | (47.8;100.0) | (54.1;100.0) | (0.8;90.6) |
|  | V6 (D57) | n | 26 | 26 | 27 | 28 | 2 | 5 | 6 | 0 |
|  |  | Percentage | 89.7 | 86.7 | 93.1 | 96.6 | 6.9 | 100.0 | 100.0 | 0.0 |
|  |  | 95% CI in %* | (72.6;97.8) | (69.3;96.2) | (77.2;99.2) | (82.2;99.9) | (0.8;22.8) | (47.8;100.0) | (54.1;100.0) | - |
|  | V8 (D85) | n | 27 | 25 | 28 | 27 | 4 | 5 | 6 | 0 |
|  |  | Percentage | 93.1 | 83.3 | 96.6 | 93.1 | 13.8 | 100.0 | 100.0 | 0.0 |
|  |  | 95% CI in %* | (77.2;99.2) | (65.3;94.4) | (82.2;99.9) | (77.2;99.2) | (3.9;31.7) | (47.8;100.0) | (54.1;100.0) | - |
|  | V9 (EOS D225) | n | 28 | 25 | 25 | 27 | 4 | 5 | 6 | 0 |
|  |  | Percentage | 96.6 | 83.3 | 86.2 | 93.1 | 13.8 | 100.0 | 100.0 | 0.0 |
|  |  | 95% CI in %* | (82.2;99.9) | (65.3;94.4) | (68.3;96.1) | (77.2;99.2) | (3.9;31.7) | (47.8;100.0) | (54.1;100.0) | - |
| O2a | V5 (D29) | n | 18 | 22 | 25 | 28 | 0 | 4 | 6 | 0 |
|  |  | Percentage | 62.1 | 73.3 | 86.2 | 96.6 | 0.0 | 80.0 | 100.0 | 0.0 |
|  |  | 95% CI in %* | (42.3;79.3) | (54.1;87.7) | (68.3;96.1) | (82.2;99.9) | - | (28.4;99.5) | (54.1;100.0) | - |
|  | V6 (D57) | n | 22 | 23 | 24 | 27 | 0 | 4 | 6 | 0 |
|  |  | Percentage | 75.9 | 76.7 | 82.8 | 93.1 | 0.0 | 80.0 | 100.0 | 0.0 |
|  |  | 95% CI in %* | (56.5;89.7) | (57.7;90.1) | (64.2;94.2) | (77.2;99.2) | - | (28.4;99.5) | (54.1;100.0) | - |
|  | V8 (D85) | n | 24 | 22 | 24 | 26 | 1 | 4 | 6 | 0 |
|  |  | Percentage | 82.8 | 73.3 | 82.8 | 89.7 | 3.4 | 80.0 | 100.0 | 0.0 |
|  |  | 95% CI in %* | (64.2;94.2) | (54.1;87.7) | (64.2;94.2) | (72.6;97.8) | (0.1;17.8) | (28.4;99.5) | (54.1;100.0) | - |
|  | V9 (EOS D225) | n | 22 | 22 | 22 | 26 | 2 | 4 | 6 | 0 |
|  |  | Percentage | 75.9 | 73.3 | 75.9 | 89.7 | 6.9 | 80.0 | 100.0 | 0.0 |
|  |  | 95% CI in %* | (56.5;89.7) | (54.1;87.7) | (56.5;89.7) | (72.6;97.8) | (0.8;22.8) | (28.4;99.5) | (54.1;100.0) | - |
| O2afg | V5 (D29) | n | 17 | 22 | 23 | 27 | 0 | 5 | 5 | 0 |
|  |  | Percentage | 58.6 | 73.3 | 79.3 | 93.1 | 0.0 | 100.0 | 83.3 | 0.0 |
|  |  | 95% CI in %* | (38.9;76.5) | (54.1;87.7) | (60.3;92.0) | (77.2;99.2) | - | (47.8;100.0) | (35.9;99.6) | - |
|  | V6 (D57) | n | 20 | 23 | 26 | 27 | 2 | 5 | 2 | 0 |
|  |  | Percentage | 69.0 | 76.7 | 89.7 | 93.1 | 6.9 | 100.0 | 33.3 | 0.0 |
|  |  | 95% CI in %* | (49.2;84.7) | (57.7;90.1) | (72.6;97.8) | (77.2;99.2) | (0.8;22.8) | (47.8;100.0) | (4.3;77.7) | - |
|  | V8 (D85) | n | 21 | 26 | 25 | 27 | 5 | 5 | 4 | 0 |
|  |  | Percentage | 72.4 | 86.7 | 86.2 | 93.1 | 17.2 | 100.0 | 66.7 | 0.0 |
|  |  | 95% CI in %* | (52.8;87.3) | (69.3;96.2) | (68.3;96.1) | (77.2;99.2) | (5.8;35.8) | (47.8;100.0) | (22.3;95.7) | - |
|  | V9 (EOS D225) | n | 24 | 23 | 25 | 26 | 5 | 4 | 2 | 0 |
|  |  | Percentage | 82.8 | 76.7 | 86.2 | 89.7 | 17.2 | 80.0 | 33.3 | 0.0 |
|  |  | 95% CI in %* | (64.2;94.2) | (57.7;90.1) | (68.3;96.1) | (72.6;97.8) | (5.8;35.8) | (28.4;99.5) | (4.3;77.7) | - |
| O3b | V5 (D29) | n | 7 | 7 | 7 | 12 | 0 | 3 | 0 | 0 |
|  |  | Percentage | 24.1 | 23.3 | 24.1 | 41.4 | 0.0 | 60.0 | 0.0 | 0.0 |
|  |  | 95% CI in %* | (10.3;43.5) | (9.9;42.3) | (10.3;43.5) | (23.5;61.1) | - | (14.7;94.7) | - | - |
|  | V6 (D57) | n | 8 | 9 | 8 | 14 | 1 | 0 | 0 | 0 |
|  |  | Percentage | 27.6 | 30.0 | 27.6 | 48.3 | 3.4 | 0.0 | 0.0 | 0.0 |
|  |  | 95% CI in %* | (12.7;47.2) | (14.7;49.4) | (12.7;47.2) | (29.4;67.5) | (0.1;17.8) | - | - | - |
|  | V8 (D85) | n | 12 | 16 | 10 | 15 | 5 | 0 | 1 | 0 |
|  |  | Percentage | 41.4 | 53.3 | 34.5 | 51.7 | 17.2 | 0.0 | 16.7 | 0.0 |
|  |  | 95% CI in %* | (23.5;61.1) | (34.3;71.7) | (17.9;54.3) | (32.5;70.6) | (5.8;35.8) | - | (0.4;64.1) | - |
|  | V9 (EOS D225) | n | 10 | 10 | 8 | 14 | 5 | 2 | 0 | 0 |
|  |  | Percentage | 34.5 | 33.3 | 27.6 | 48.3 | 17.2 | 40.0 | 0.0 | 0.0 |
|  |  | 95% CI in %* | (17.9;54.3) | (17.3;52.8) | (12.7;47.2) | (29.4;67.5) | (5.8;35.8) | (5.3;85.3) | - | - |

Table S5. Summary data of GMT for serum IgG against the different O serotypes: fold increase from baseline – population 55-70 years (IAS)

|  | | | | | | | |
| --- | --- | --- | --- | --- | --- | --- | --- |
|  |  |  | **Treatment** | | | | |
| **Serotype** | **Visit** |  | **Kleb4V 16µg (N=29)** | **Kleb4V 16µg + AS03 (N=30)** | **Kleb4V 64µg (N=29)** | **Kleb4V 64µg + AS03 (N=29)** | **Placebo (N=29)** |
| O1v1 | V4 (D15) | n | 23 | 23 | 23 | 23 | 21 |
|  |  | Geometric Mean | 30.410 | 27.907 | 57.293 | 75.109 | 0.985 |
|  |  | 95% CI | (12.640;73.162) | (11.811;65.937) | (27.143;120.931) | (30.128;187.246) | (0.743;1.307) |
|  | V5 (D29) | n | 29 | 28 | 29 | 28 | 29 |
|  |  | Geometric Mean | 43.987 | 44.240 | 66.918 | 80.403 | 0.920 |
|  |  | 95% CI | (21.426;90.307) | (20.576;95.116) | (31.977;140.041) | (36.367;177.760) | (0.857;0.987) |
|  | V6 (D57) | n | 29 | 29 | 29 | 29 | 29 |
|  |  | Geometric Mean | 54.904 | 48.626 | 104.700 | 58.123 | 1.155 |
|  |  | 95% CI | (27.257;110.593) | (23.477;100.714) | (53.200;206.057) | (27.075;124.774) | (0.773;1.725) |
|  | V8 (D85) | n | 29 | 29 | 29 | 29 | 29 |
|  |  | Geometric Mean | 54.565 | 40.439 | 79.307 | 64.895 | 1.684 |
|  |  | 95% CI | (26.147;113.868) | (19.240;84.997) | (44.260;142.105) | (33.159;127.008) | (1.103;2.570) |
|  | V9 (EOS D225) | n | 29 | 29 | 28 | 29 | 29 |
|  |  | Geometric Mean | 33.655 | 22.919 | 40.544 | 28.117 | 1.988 |
|  |  | 95% CI | (18.683;60.625) | (11.432;45.949) | (20.303;80.966) | (11.699;67.573) | (1.157;3.416) |
| O2a | V4 (D15) | n | 23 | 23 | 23 | 23 | 21 |
|  |  | Geometric Mean | 9.912 | 12.113 | 18.286 | 47.678 | 0.865 |
|  |  | 95% CI | (4.016;24.464) | (6.295;23.309) | (8.028;41.651) | (26.338;86.309) | (0.714;1.049) |
|  | V5 (D29) | n | 29 | 28 | 29 | 28 | 29 |
|  |  | Geometric Mean | 13.513 | 21.281 | 18.533 | 40.380 | 0.922 |
|  |  | 95% CI | (6.553;27.865) | (10.767;42.060) | (9.878;34.774) | (26.239;62.143) | (0.823;1.033) |
|  | V6 (D57) | n | 29 | 29 | 29 | 29 | 29 |
|  |  | Geometric Mean | 14.420 | 16.588 | 19.613 | 23.410 | 0.965 |
|  |  | 95% CI | (7.703;26.992) | (9.054;30.391) | (10.193;37.736) | (13.297;41.215) | (0.702;1.326) |
|  | V8 (D85) | n | 29 | 29 | 29 | 29 | 29 |
|  |  | Geometric Mean | 15.954 | 21.837 | 18.078 | 31.910 | 1.277 |
|  |  | 95% CI | (8.247;30.863) | (10.115;47.142) | (9.031;36.185) | (20.186;50.444) | (0.900;1.813) |
|  | V9 (EOS D225) | n | 29 | 29 | 28 | 29 | 29 |
|  |  | Geometric Mean | 12.776 | 10.724 | 11.448 | 20.547 | 1.566 |
|  |  | 95% CI | (7.431;21.964) | (5.517;20.845) | (5.202;25.194) | (10.968;38.490) | (1.041;2.356) |
| O2afg | V4 (D15) | n | 23 | 23 | 23 | 23 | 21 |
|  |  | Geometric Mean | 8.063 | 11.294 | 20.928 | 41.927 | 0.672 |
|  |  | 95% CI | (3.233;20.105) | (5.574;22.882) | (11.165;39.227) | (18.073;97.263) | (0.509;0.886) |
|  | V5 (D29) | n | 29 | 28 | 29 | 28 | 29 |
|  |  | Geometric Mean | 13.448 | 11.488 | 17.156 | 50.278 | 0.872 |
|  |  | 95% CI | (6.392;28.293) | (6.979;18.911) | (9.470;31.082) | (25.436;99.380) | (0.756;1.005) |
|  | V6 (D57) | n | 29 | 29 | 29 | 29 | 29 |
|  |  | Geometric Mean | 11.508 | 9.894 | 26.850 | 39.467 | 1.117 |
|  |  | 95% CI | (5.644;23.466) | (5.306;18.451) | (14.511;49.679) | (20.383;76.419) | (0.794;1.572) |
|  | V8 (D85) | n | 29 | 29 | 29 | 29 | 29 |
|  |  | Geometric Mean | 15.908 | 22.499 | 25.583 | 53.199 | 1.408 |
|  |  | 95% CI | (7.653;33.066) | (12.155;41.643) | (13.383;48.904) | (30.253;93.548) | (0.884;2.244) |
|  | V9 (EOS D225) | n | 29 | 29 | 28 | 29 | 29 |
|  |  | Geometric Mean | 17.153 | 10.806 | 21.971 | 39.899 | 2.100 |
|  |  | 95% CI | (9.616;30.597) | (5.804;20.119) | (12.034;40.114) | (16.166;98.472) | (1.057;4.171) |
| O3b | V4 (D15) | n | 23 | 23 | 23 | 23 | 21 |
|  |  | Geometric Mean | 1.987 | 1.917 | 1.943 | 5.298 | 0.778 |
|  |  | 95% CI | (1.277;3.089) | (1.146;3.206) | (1.194;3.164) | (3.116;9.008) | (0.613;0.987) |
|  | V5 (D29) | n | 29 | 28 | 29 | 28 | 29 |
|  |  | Geometric Mean | 2.412 | 2.398 | 2.182 | 4.501 | 0.963 |
|  |  | 95% CI | (1.683;3.457) | (1.677;3.428) | (1.596;2.982) | (2.942;6.887) | (0.925;1.001) |
|  | V6 (D57) | n | 29 | 29 | 29 | 29 | 29 |
|  |  | Geometric Mean | 2.071 | 2.485 | 2.559 | 4.594 | 0.878 |
|  |  | 95% CI | (1.373;3.125) | (1.567;3.941) | (1.639;3.996) | (2.952;7.150) | (0.636;1.214) |
|  | V8 (D85) | n | 29 | 29 | 29 | 29 | 29 |
|  |  | Geometric Mean | 3.390 | 3.787 | 2.737 | 5.240 | 1.136 |
|  |  | 95% CI | (1.786;6.435) | (2.244;6.390) | (1.578;4.747) | (2.763;9.939) | (0.765;1.685) |
|  | V9 (EOS D225) | n | 29 | 29 | 28 | 29 | 29 |
|  |  | Geometric Mean | 3.169 | 2.688 | 1.852 | 3.959 | 1.578 |
|  |  | 95% CI | (2.089;4.807) | (1.639;4.408) | (1.082;3.169) | (2.064;7.595) | (1.044;2.386) |
